# Effect of Excess Iodide Intake on Salivary Glands in a Swiss Albino Mice Model

**DOI:** 10.1155/2017/6302869

**Published:** 2017-11-08

**Authors:** Gloria Romina Ross, Emanuel Fabersani, Matías Russo, Alba Gómez, Hugo Japaze, Silvia Nelina González, Paola Gauffin Cano

**Affiliations:** ^1^Instituto de Biotecnología Farmacéutica y Alimentaria (INBIOFAL-CONICET), Tucumán, Argentina; ^2^Facultad de Ciencias de la Salud, Universidad del Norte Santo Tomás de Aquino (UNSTA), Tucumán, Argentina; ^3^Facultad de Agronomía y Zootecnia, Universidad Nacional de Tucumán, Tucumán, Argentina; ^4^Centro de Referencia para Lactobacilos (CERELA-CONICET), Tucumán, Argentina; ^5^Facultad de Medicina, Universidad Nacional de Tucumán, Tucumán, Argentina

## Abstract

Iodine is an important micronutrient required for nutrition. Excess iodine has adverse effects on thyroid, but there is not enough information regarding its effect on salivary glands. In addition to food and iodized salt, skin disinfectants and maternal nutritional supplements contain iodide, so its intake could be excessive during pregnancy, lactation, and infancy. The aim of this work was to evaluate the effect of excess iodide ingestion on salivary glands during mating, gestation, lactation, and postweaning period in mouse. During assay, mice were allocated into groups: control and treatment groups (received distilled water with NaI 1 mg/mL). Water intake, glandular weight, and histology were analyzed. Treatment groups showed an increase in glandular weight and a significantly (*p* < 0.05) higher water intake than control groups. Lymphocyte infiltration was observed in animals of treatment groups, while there was no infiltration in glandular sections of control groups. Results demonstrated that a negative relationship could exist between iodide excess and salivary glands. This work is novel evidence that high levels of iodide intake could induce mononuclear infiltration in salivary glands. These results should be considered, especially in pregnant/lactating women, to whom a higher iodine intake is usually recommended.

## 1. Introduction

Iodine is an important micronutrient required for human nutrition; it is an essential component of thyroid hormones (TH). These hormones are necessary for normal growth and differentiation of cells, fetal growth, nervous system, reproductive tract development, bone formation, and so forth [1].

Iodine concentration in water is an important index of human's natural iodine intake [2]. Recommended Dietary Allowances (RDAs) represent the average daily level of intake sufficient to meet the nutrient requirements of nearly all (97%-98%) healthy individuals. RDAs for iodine vary by age and gender. For a healthy adult, RDAs value for iodine is 150 mcg to maintain a steady state between uptake and secretion of hormones from the thyroid. During pregnancy and lactation, slightly higher iodine intakes per day are recommended (220 mcg and 290 mcg, respectively) [3]. Iodine deficiency leads to several disorders which are referred to as iodine deficiency disorders (IDD). Among them are endemic goiter, stillbirth, mental retardation, deaf mutism, and cretinism among young children [4, 5].

The global effort to prevent IDD through iodine supplementation, such as universal salt iodization (USI), has achieved impressive progress during the last few decades [6]. Iodination of salt, by addition of potassium iodate, is a strategy recommended by the United Nations Children's Fund (UNICEF) and the World Health Organization (WHO), as a public health measure [7]. Due to supplementing iodine in ordinary table salt, iodine intake levels have greatly increased worldwide; however, at present there are problems related to overconsumption of dietary iodine [8].

There are several factors involved in excess iodine: high levels of salt iodization and overlapping iodine supplementation, iodine-rich drinking water as well as iodine-rich foods intake (kelp and seaweed, iodine additives to bread/flour, preservatives, red coloring, etc.) [6, 9]. Nowadays, there is insufficient evidence that excess iodine can also have adverse effects depending on underlying thyroid function, as well as the extent and duration of iodine excess [10]. It was demonstrated that high iodine intake involved risks such hypothyroidism, hyperthyroidism, development of goiter, and cancer [5, 11, 12]. These diseases caused, in vulnerable people, thyroid destruction and hence presentation of thyroidal antigens to the immune system leading to an autoimmune reaction and the development of autoimmune thyroid disease (AITD) [13]. There is a correlation between iodine intake and harshness of disease [7].

Although there are numerous studies that show the effects of excess iodine ingestion on thyroids, using murine models that closely resembles human disease, there are not enough information regarding the effect of high levels of iodine intake on salivary glands. The salivary glands, like thyroids, are able to concentrate iodide so its salivary concentration is from 20 to 100 times more than that found in the serum. This glandular critical ability could cause damage, like cellular infiltration, in this extrathyroid tissue [14, 15]. Lymphocyte accumulation in salivary glands represents one of the leading causes of dry eye and mouth in the world, with these symptoms being also related to Sjogren syndrome, a systemic chronic autoimmune disease that targets predominantly the salivary glands and lacrimal glands [16]. On the other hand, during pregnancy, lactation, and infancy, iodide recommended intake increases because it is known that iodide deficiency may cause irreversible cognitive impairment [17]. However, it is very important to know that, in addition to food and iodized salt, skin disinfectants and maternal nutritional supplements contain iodide, so its intake could be excessive in these periods of life. Thus, the aim of this work was to study the effect of excessive iodine intakes on salivary glands, during mating, pregnancy, lactation, and postweaning period on a murine animal model.

## 2. Materials and Methods

### 2.1. Animals and Treatment

Animals were acquired from the closed random-bred colony kept at Centro de Referencia para Lactobacilos (CERELA-CONICET). They were maintained in individual cages and acclimated to 22 ± 2°C with a 12 h light/dark cycle. Throughout the assay, they received standard commercial food, meeting the nutritional requirements of animals (61% carbohydrates, 23% proteins, 7.5% fats, 4% raw fiber, 3.5% total minerals (3.10 Kcal/g); Asociación de Cooperativas Argentinas, Buenos Aires, Argentina), and distilled water ad libitum.

The experimental protocol was designed in two phases.

**Phase 2.1 I.**

Before mating until weaning of offspring (mating, gestation, and lactation), 20 female Swiss albino mice (6–8 weeks of age) were randomly allocated into two groups, receiving the following during 42 days:

- control group (*n* = 10): distilled water [18–22],
- treatment group (*n* = 10): distilled water supplemented with NaI 1 mg/mL.

At the end of this phase, body weights were measured. Animals were anesthetized (100 mg/kg ketamine and 10 mg/kg xylazine via intraperitoneal injection) prior to removing and weighing salivary glands. Animals were sacrificed by cervical dislocation.

**Phase 2.1 II.**

After weaning (21 days of age), breeding mice were separated from mother. This phase was carried out with female offspring, as there is evidence that salivary gland disorders are more predominant in females [23–25]

Female offspring were also allocated into two groups, receiving the following during 35 days:

- control group (*n* = 18, murine offspring from control group): distilled water,
- treatment group (*n* = 18, murine offspring from treatment group) distilled supplemented water with NaI 1 mg/mL.

Throughout experimental Phases I and II, water intake was monitored weekly and is expressed as mL consumed per mouse per day.

### 2.2. Samples Preparation

At days 0, 14, and 35, animals were weighed and anesthetized (100 mg/kg ketamine and 10 mg/kg xylazine via intraperitoneal injection). Animal's neck was cut up at midline to harvest salivary glands. Glandular tissue was washed with saline buffer, dried on a filter paper, and weighed using an electronic balance (Traveler TA302, Ohaus Corporation, USA). Finally, animals were sacrificed by cervical dislocation.

All experimental procedures were approved by the Animal Protection Committee of CERELA and complied with current Argentinean laws.

### 2.3. Histological Analysis

The salivary gland tissue was fixed in 10% formaldehyde and embedded in paraffin. Sections (3 *μ*m) were stained with hematoxylin and eosin (HE) for histological analysis. Pathological changes in the glandular tissue were investigated under light microscopy (Carl Zeiss Axio Scope A1, Gottingen, Germany).

Histological salivary tissues were scored as follows: Grade 0, tissues without mononuclear infiltration; Grade 1, tissues with dispersed lymphocytic infiltration; Grade 2, with moderate infiltration; Grade 3 with one or more focus groups of 50 or more lymphocytes [26].

### 2.4. Statistical Analysis

Results were expressed as mean ± standard deviation (SD). Data were analyzed with one-way ANOVA using SPSS version 12.0 (SPSS Inc., Chicago, IL, USA). Differences were considered significant at *p* < 0.05 using Tukey's test.

## 3. Results and Discussion

### 3.1. Percentage of Relative Glandular Weight

The percentage of relative salivary gland weight (salivary gland weight (g)/100 g mouse body weight) was determinate at the end of Phase I (42 days) and at 0, 14, and 35 days of Phase II (Table 1).

Throughout Phase II, for both control and treatment groups, relative glandular weights increase gradually with growth and age (Table 1).

Table 1 shows that relative glandular weights of animals from treatment group were slightly greater than those of animals from control groups, during both Phases I and II. However, the increases observed were not significant (*p* < 0.05).

We have not found information that shows the relationship between iodide intake and salivary gland weights, but there are studies that demonstrated a positive correlation between iodide consumption and thyroid weight in NOD.H-2h4 mice [27] and in guinea pigs [28]. Thyroid is a glandular tissue situated in the anterior region of the neck; it is one of the endocrine active organs. Its embryogenesis is closely related to the gastrointestinal tract, showing the same functions and abilities to metabolize iodide and to actively accumulate it as the salivary and gastric glands [29, 30]. In addition, the Na (+)/I (−) symporter (NIS) is an integral plasma membrane glycoprotein that mediates active iodide transport into the thyroid follicular cells and also mediates active iodide transport in other tissues, including salivary glands, gastric mucosa, and lactating mammary gland [31, 32]. Teng et al. [27] show that as the iodine intake increased, the weight of the thyroid gradually increased as well in a murine model. They found a significantly positive correlation between relative thyroid weight (thyroid weight/mouse body weight) and the dosage of iodine. Based on these findings, the increase in related salivary gland weights of animals from treatment groups induced by iodide water intake could be explained.

### 3.2. Water Intake Measurements

Throughout experimental Phases I and II, water intake was monitored weekly and is expressed as mL water consumed per mouse per day (Table 2)

During Phase I, there were not significant differences (*p* < 0.05) in the water intake between animals of control and treatment groups until the fifth week of the assay. At the sixth week, water consumption by animals of treatment group was significantly higher compared with control group (*p* < 0.05) (Table 2)

Regarding Phase II, water intake consumed by animals of treatment group increased significantly (*p* < 0.05) compared to control group over the 5-week experimental period (Table 2).

The results show that prolonged excess iodine intake would be related to higher water consumption by animals of treatment group. There is evidence that excess iodine induced lymphocytic infiltration in different tissues, such as thyroid [33] and salivary gland [34, 35], and could consequently cause hypofunction of these tissues. Regard salivary glands, loss of salivary function induces oral dryness (xerostomia) [36–38]. It is well established that after ingestion of high levels of sodium, there is a subsequent rise in plasma sodium, and to maintain fluid homeostasis, thirst is stimulated. However sodium stimulated thirst rapidly, compared with the highest water intake related to symptoms of dryness caused by infiltration in salivary gland [39]. For this reason, significant gradual increase in water intake consumed by animals from treatment groups would be explained, as iodine intake gets higher.

### 3.3. Histological Analysis

**Figure 1:**
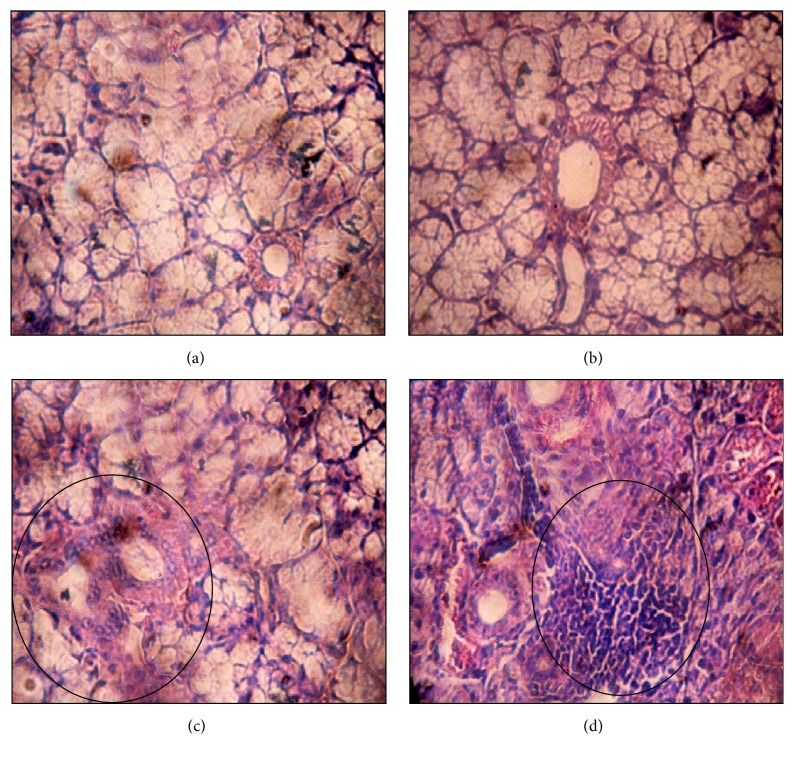
Hematoxylin and eosin (H&E 40x) stained salivary glands sections. (a) Control group (Phase I). (b) Control group (Phase II). (c) Treatment group (histological score = Grade 2). (d) Treatment group (histological score = Grade 3). Lymphocytic infiltrations are surrounded by circles.

Histological examination demonstrated normal salivary gland histology in most of animals from control groups (Phases I and II).  Table 3 shows that more than 88% of salivary tissues from control groups presented conserved structures in addition to the absence of cell infiltration (Grade 0) (Figure 1(a)).

Instead, most glandular tissues from treatment groups revealed focal lymphocytic infiltration. Mononuclear follicles were disposed mainly around salivary ducts. At the end of Phase I, 60% and 20% of tissue samples showed dispersed (Grade 1) and moderate (Grade 2) lymphocytic infiltration, respectively (Figure 1(c)). Regarding Phase II, tissues revealed moderate infiltration (44.4%) and presence of focus in the neighborhood of salivary gland ducts (38.9%) in particular (Figure 1(d)). Results are in accordance with Takegawa et al. [34] and S. Venturi and M. Venturi [35] who demonstrated that high levels of iodine induced lymphocytic infiltration in tissues such as thyroid and salivary gland.

## 4. Conclusions

Several researches revealed that large amounts of iodine given for days to months have shown few adverse effects on thyroid and extrathyroid tissues [10], demonstrating that there is a positive iodine dose-damage relationship [40]. The results shown in this work indicate that there could be a correlation between extent and duration of iodine excess intake and negative effects on salivary glands. Throughout Phase I, side effects of iodine excess intake on salivary glands were not observed, probably due to the short period of iodine excess ingestion by animals. Significant differences in water consumption by animals of treatment group were only observed, during the last week of Phase I and during Phase II, so oral dryness due to loss of salivary function could be associated with a prolonged excess iodine intake.

The results obtained in this work show that excess iodine ingestion from mating until postweaning period, in a mice model, induced various degrees of inflammatory cell infiltration in salivary glands. Cell infiltration found in animals that received high levels of iodine could be associated with Sjogren syndrome (SS). This syndrome is a chronic and progressive systemic autoimmune disease that primarily involves immune-mediated damage to the lacrimal and salivary glands. SS is the second most prevalent autoimmune disease, affecting about four million Americans, especially women. The symptoms of SS usually progress slowly and are often highly variable in presentation, making diagnosis difficult [39]. Although there have been no reports of Sjogren syndrome in mouse model treated with NaI, there are some references that support the possibility that chemicals containing iodine impair salivary gland function in man and rats [41]. More studies are warranted to effectively diagnose Sjogren syndrome, as positive serum anti-SSA/Ro and/or anti-SSB/La, and keratoconjunctivitis sicca.

Based on this work salivary gland could be considered a target organ of compounds containing iodine, so it will be necessary to regulate the supplementing of iodide, especially during pregnancy and lactation, to avoid overconsumption.

## Figures and Tables

**Table 1:** Percentage of relative glandular weight of Swiss albino mice at Phases I and II.

Day	Salivary gland weight (g)/mouse body weight (100 g)	
	Control group	Treatment group
	Phase I	
42	0.62 ± 0.03^a^	0.68 ± 0.08^a^

	Phase II	
0	0.53 ± 0.02^a^	0.56 ± 0.04^a^
14	0.56 ± 0.10^a^	0.63 ± 0.10^a^
35	0.62 ± 0.03^a^	0.70 ± 0.08^a^

Percentage of relative weight of salivary gland denotes glandular weight (g)/mouse body weight (100 g). Results are represented as mean ± SD and expressed as g. Different letters in the same row indicate significant differences (*p* < 0.05).

**Table 2:** Water intake (mL/mouse/day) during experimental Phases I and II.

Week	Water intake (mL/mouse/day)			
	Phase I		Phase II	
	Control group	Treatment group	Control group	Treatment group
1	3.7 ± 0.2^a^	3.5 ± 0.3^a^	3.2 ± 0.2^a^	3.7 ± 0.1^b^
2	3.5 ± 0.3^a^	3.6 ± 0.2^a^	3.8 ± 0.1^a^	4.9 ± 0.2^b^
3	3.9 ± 0.2^a^	3.7 ± 0.2^a^	3.6 ± 0.3^a^	6.6 ± 0.4^b^
4	4.3 ± 0.3^a^	4.8 ± 0.4^a^	4.1 ± 0.2^a^	7.3 ± 0.2^b^
5	4.5 ± 0.2^a^	4.7 ± 0.1^a^	4.2 ± 0.4^a^	9.4 ± 0.2^b^
6	4.8 ± 0.1^a^	5.6 ± 0.2^b^	—	—

Results are represented as mean ± SD and expressed as (mL/mouse/day). Phase I: different letters in the same row indicate significant differences (*p* < 0.05). Phase II: different letters in the same row indicate significant differences (*p* < 0.05).

**Table 3:** Distribution of salivary tissues according to histological score.

Grade	Histological score			
	Phase I		Phase II	
	Control group *n* (%)	Treatment group *n* (%)	Control group *n* (%)	Treatment group *n* (%)
0	9 (90)	2 (20)	16 (88.8)	1 (5.6)
1	1 (10)	6 (60)	1 (5.6)	2 (11.1)
2		2 (20)	1 (5.6)	8 (44.4)
3				7 (38.9)
*Total*	*10 (100)*	*10 (100)*	*18 (100)*	*18 (100)*
